# Clinical features of the pathogenic m.5540G>A mitochondrial transfer RNA tryptophan gene mutation

**DOI:** 10.1016/j.nmd.2016.08.009

**Published:** 2016-10

**Authors:** Yi Shiau Ng, Steven A. Hardy, Venice Shrier, Gerardine Quaghebeur, David R. Mole, Matthew J. Daniels, Susan M. Downes, Jane Freebody, Carl Fratter, Monika Hofer, Andrea H. Nemeth, Joanna Poulton, Robert W. Taylor

**Affiliations:** a Wellcome Trust Centre for Mitochondrial Research, Institute of Neuroscience, Newcastle University, Newcastle upon Tyne, UK; b Nuffield Department of Obstetrics and Gynaecology, Women's Centre, Oxford, UK; c Department of Neuroradiology, Oxford University Hospitals NHS Foundation Trust, Oxford, UK; d Oxford Kidney Unit, Oxford University Hospitals NHS Foundation Trust, Oxford, UK; e Division of Cardiovascular Medicine, BHF Oxbridge Centre of Regenerative Medicine, Oxford University, Oxford, UK; f Oxford Eye Hospital, Oxford, UK; g Neurosciences Offices, Level 3 West Wing, John Radcliffe Hospital, Oxford University Hospitals NHS Foundation Trust, Oxford, UK; h Oxford Medical Genetics Laboratories, Oxford University Hospitals NHS Trust, Churchill Hospital, Oxford, UK; i Neuropathology and Ocular Pathology Department, John Radcliffe Hospital, Oxford, UK; j Nuffield Department of Clinical Neurosciences, University of Oxford, Oxford, UK; k Churchill Hospital, Oxford, UK

**Keywords:** Mitochondrial DNA disease, Ataxia, Muscle biopsy, Proteinuria, Stroke

## Abstract

Mitochondrial DNA disease is one of the most common groups of inherited neuromuscular disorders and frequently associated with marked phenotypic and genotypic heterogeneity. We describe an adult patient who initially presented with childhood-onset ataxia without a family history and an unremarkable diagnostic muscle biopsy. Subsequent multi-system manifestations included basal ganglia calcification, proteinuria, cataract and retinitis pigmentosa, prompting a repeat muscle biopsy that showed features consistent with mitochondrial myopathy 13 years later. She had a stroke with restricted diffusion change in the basal ganglia and internal capsule at age 44 years. Molecular genetic testing identified a previously-reported pathogenic, heteroplasmic mutation in the mitochondrial-encoded transfer RNA tryptophan (*MT-TW*) gene which based on family studies was likely to have arisen *de novo* in our patient. Interestingly, we documented an increase in the mutant mtDNA heteroplasmy level in her second biopsy (72% compared to 56%), reflecting the progression of clinical disease.

## Highlights

• Longitudinal increase in mtDNA mutant load reflects worsening muscle histochemistry.
• *De novo* m.5540G>A mtDNA mutation adds to its credentials as a pathogenic mutation.
• Additional clinical findings are cataract, kidney disease and stroke.

## 1 Introduction

Mitochondrial DNA (mtDNA) disease is clinically heterogeneous with variable age of onset ranging from fatal, infantile-onset presentations such as Leigh syndrome to late-adult onset chronic progressive external ophthalmoplegia (CPEO). Although neurological features are prominent in most cases, there is often multi-system involvement [1], [2]. Since the first pathogenic mutations were identified in the mitochondrial genome in 1988, more than 300 different single nucleotide variants have been reported in association with human disease. Whilst common point mutations such as the m.3243A>G mutation and three primary Leber hereditary optic neuropathy (LHON) mutations are prevalent in the population and have been extensively studied [3], novel and/or rare pathogenic variants are often reported in single or small numbers of family pedigrees and therefore the spectrum of phenotype and severity is poorly understood.

The m.5540G>A mutation in the mitochondrial (mt-) tRNA^Trp^ (*MT-TW*) gene has been associated with refractory epilepsy, ataxia, neuropathy, cognitive impairment, myopathy and pigmentary retinopathy [4], [5]. Here we report an adult patient who harbours this mutation but displays additional clinical features that have not been previously described.

## 2 Patient and methods

### 2.1 Case report

A 45 year old Caucasian woman had a long history of falls due to progressive ataxia since the age of 10. Her birth history and early developmental milestones were unremarkable. She completed secondary school and achieved basic qualification in education but was never good in sport. At age 17, she developed poor night vision and bilateral hearing deficit. She had bilateral cataract surgeries and used hearing aids at age 31. She experienced fatigue but did not complain of overt muscle weakness. She developed moderately heavy proteinuria (Albumin to creatinine ratio 152) but with normal serum albumin level since age 43.

Her mother has glucose intolerance and her father has sensorineural deafness. There was no family history of neurological disorder, eye problem, or cardiomyopathy. All her siblings are fit and well, and as such did not request molecular genetic testing.

**Fig. 1:**
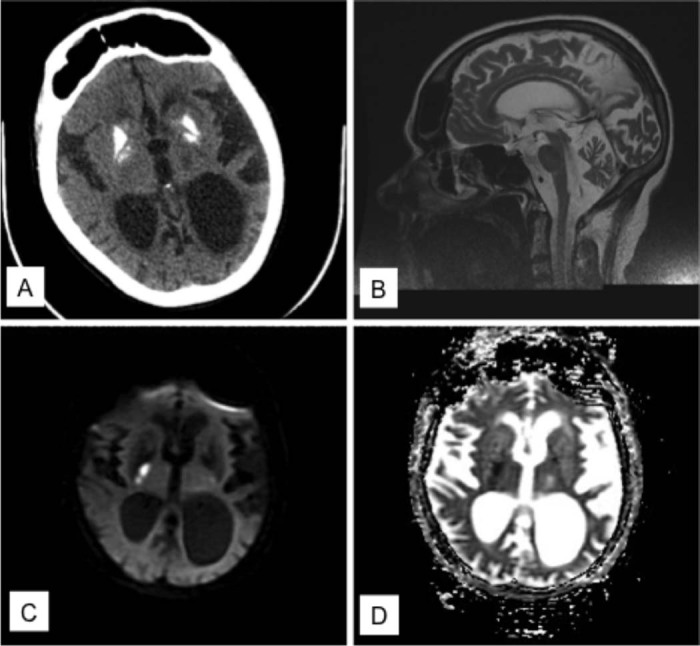
**Neuroimaging results** (A) CT head showed bilateral, symmetrical basal ganglia calcification. (B) Sagittal view of cranial MRI showed significant cerebral and cerebellar atrophy at the age of 39 years. (C) Diffusion-weighted imaging showed restricted diffusion in the right internal capsule and lentiform nucleus with corresponding low signal change in (D) ADC map at the age of 44 years.

Her resting blood lactate level was normal and creatine kinase (CK) level was 276 U/L. There was no glucose intolerance. Her renal function declined gradually and the current urea and creatinine levels were 11 mmol/L and 97 µmol/L, respectively (compared to 5 mmol/L and 88 µmol/L 8 years ago). Renal ultrasound demonstrated loss of cortico-medullary differentiation, increased echogenicity and asymmetrical kidney size (left kidney measured 7.9 cm, right kidney 10.2 cm). Nerve conduction studies were normal at age 28. Her initial CT head and cranial MRI showed bilateral basal ganglia calcification, periventricular white matter changes and cerebral and cerebellar atrophy (Fig. 1A and B).

Clinical examination (at age 43) revealed pigmentary retinopathy, deafness, bilateral pes cavus, areflexia in the lower limbs, mild myopathy with MRC grade 4+/5, cerebellar signs, loss of proprioception sense and vibration sense in the lower limbs and broad-based gait.

She was admitted to hospital with left sided weakness and loss of mobility at age 44. The neurological deficit evolved over 12 hours. There was no associated speech disturbance or visual field defect. Her ECG showed sinus rhythm and she had hypertension. Cranial MRI revealed acute changes involving the right internal capsule and lentiform nucleus (Fig. 1C and D). She was treated with antiplatelet, angiotensin converting enzyme inhibitor, oral L-arginine and co-enzyme Q10. Further cardiac assessment including 48 hour ambulatory ECG and transthoracic echocardiogram was unremarkable. Despite a period of rehabilitation, she only made partial recovery and required walking aid for mobility. In addition, the cerebellar syndrome has progressed with worsening dysarthria and ataxia on the last clinic review.

### 2.2 Histopathology, biochemistry and molecular genetic studies

This patient underwent two muscle biopsies, the first at the age of 28 years and a second biopsy at 41 years of age. Both muscle samples were subjected to standard histopathological analyses. Informed consent was obtained from the patient and her mother for genetic testing. Total DNA from blood (leucocytes), muscle, saliva and urine was extracted by standard procedures. Long-range PCR of muscle DNA was performed to screen samples for large-scale mtDNA rearrangements. The methods for sequencing of entire mtDNA and quantitative pyrosequencing of mtDNA heteroplasmy level have been described elsewhere [6]. In addition to the patient's samples, blood, urine and saliva samples from the patient's mother were also obtained for mitochondrial genetic studies.

## 3 Results

**Fig. 2:**
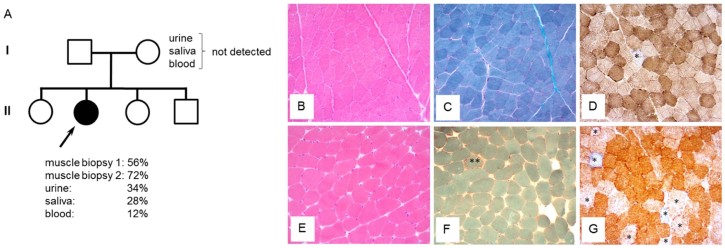
**mtDNA mutation analysis in the patient and her mother** (A) Family tree identifying the proband (highlighted by an arrow) with the m.5540G>A mutation. Analysis of several samples from the patient's clinically-unaffected mother strongly suggested that the m.5540G>A mutation has arisen *de novo*. (B–D) A first muscle biopsy, performed at the age of 28 years, shows normal muscle architecture in the H&E (B) and modified Gomori trichrome (C) stains and only occasional COX-deficient fibres (asterisked) identified following sequential COX/SDH histochemistry (D). (E–G) A second muscle biopsy, performed 13 years later, shows slightly rounded muscle fibres (E), an occasional ragged-red fibre (**) and an apparent increase in the number of COX-deficient fibres and those with diminished COX reactivity (asterisked, G).

The first muscle biopsy was initially reported as normal histopathologically, whilst respiratory chain enzyme studies also failed to show any major enzyme defect (data not shown). A repeat muscle biopsy (interval of 13 years) showed small, round fibres with tiny vacuoles consistent with lipid content (confirmed by Oil Red O staining), occasional ragged-red fibres (RRFs) together with cytochrome *c* oxidase (COX)-intermediate and COX-deficient fibres (Fig. 2). A retrospective review of the first muscle biopsy revealed subtle but definitive focal COX-deficiency (see Fig. 2D).

Screens for common mtDNA mutations including m.3243A>G, m.8344A>G, m.8993T>C/G and m.13513G>A were negative whilst long-range PCR protocols failed to detect any evidence of large-scale mtDNA deletions. Sequencing of the entire mitochondrial genome in muscle led to the identification of a previously-reported mutation in the mt-tRNA^Trp^ (*MT-TW*) gene – m.5540G>A. The mutation load was heteroplasmic and quantified by pyrosequencing at levels of 56% and 72% in the first and second muscle biopsies respectively, 12% in blood, 28% in saliva and 34% in urine DNA samples (Fig. 2A).

The m.5540G>A mutation was not detected in any samples (blood, urine and saliva) obtained from the patient's mother (Fig. 2A).

## 4 Discussion

Here we report a female patient who harboured the previously-described heteroplasmic m.5540G>A mutation and presented with a childhood-onset slowly progressive cerebellar ataxia complicated by multi-system manifestation. Similar to previous reports [4], [5], [7], our patient had sensorineural deafness, pigmentary retinopathy, cerebellar syndrome, peripheral neuropathy and bilateral basal calcification, cerebral and cerebellar atrophy. The additional clinical features in this case include: (1) early-onset, bilateral cataracts; (2) renal involvement manifesting with heavy proteinuria and hypertension without overt nephrotic syndrome; (3) acute, unilateral ischaemic changes in the basal ganglia.

Myopathy appears not to be a prominent clinical feature associated with this specific mtDNA mutation. There are a few published examples [8], [9] of a progression in the histopathological changes and the increase in the mutant mtDNA heteroplasmy level such as we demonstrated between the two diagnostic muscle biopsies. Our new data support the general idea that severity of the muscle phenotype increases with mutant load, as a result of clonal expansion [10]. Whilst it is also possible that the difference simply reflects heterogeneity in mutant load in different regions of her muscle, we are not aware of any report of such a large difference in muscle samples taken at the same time. We also found that mutant mtDNA heteroplasmy levels in other non-invasively obtained tissues were much lower than the muscle, similar to previous reports of a mutation hierarchy between mitotic and post-mitotic tissues [5], and consistent with a progressive loss of mutant mtDNA from blood [11]. We would speculate that a higher mutant heteroplasmy is present in the central nervous system given the patient has early-onset ataxia and has had a stroke. Furthermore, we believe that the m.5540G>A mutation has arisen *de novo* in our patient as it was not detected in multiple tissues taken from the patient's mother although the possibility of germline mosaicism in her mother's oocytes [12] could not be excluded. We have previously shown that the level of mutant mtDNA in blood is lower in sporadic compared with transmitted pathogenic mtDNA mutations [13]. The relatively low mutant load in blood DNA (12%) is entirely consistent with sporadic occurrence.

The mitochondrial genome is enormously susceptible to mutation [14]. Elliot et al. have estimated that the *de novo* mutation rate in ten common pathogenic mtDNA variants is 107/100,000 live births (95% CI = 87–127) [15]. More recently, a large case series shows the *de novo* frequency is up to 25% in patients with the mtDNA mutations, with a low recurrence risk [16]. Furthermore, six of the fifteen *MT-TW* pathogenic gene variants (including the m.5540G>A mutation) have been reported to occur as *de novo* mutations (Supplementary Table S1). These findings have important implications for clinical practice; first, the absence of family history of maternally-inherited multisystem disease should not preclude the testing for mtDNA mutations and second, carrier testing should be performed on at least two tissues to mitigate the risk of false negative results due to the variable segregation of mutant mtDNA heteroplasmy in different tissues. Thirdly, the risk of recurrence in further siblings is low for *de novo* mutations [16].

The presence of chronic proteinuria and renal insufficiency without electrolyte imbalance is suggestive of glomerular dysfunction, probably caused by the underlying mitochondrial defect in our patient, although renal biopsy has not been undertaken to exclude other aetiologies. Renal manifestations including Fanconi syndrome, focal segmental glomerulosclerosis and end-stage renal failure have been recognised in patients with pathogenic mitochondrial tRNA mutations although interestingly tubulopathy appears more commonly associated with primary single, large-scale deletion of mtDNA [17], [18].

Classical stroke-like episodes in mitochondrial disease typically manifest with evolving encephalopathy, focal seizure and neurological deficit with cortical and subcortical changes identified in the brain imaging [19]. Although symmetrical cystic changes in basal ganglia are common in patients with Leigh syndrome, unilateral acute changes in the deep nuclei, such as those observed in our patient, are rare. Her presentation of hemiparesis has been attributed to be a metabolic event because of the evolving nature of the clinical presentation. However, a lacunar stroke remains a possibility. L-arginine and co-enzyme Q10 were commenced during the hospital admission but we have not observed dramatic improvement of the neurological deficit.

In conclusion, our findings broaden the clinical phenotype associated with the m.5540G>A mutation which in our patient is likely to have arisen sporadically and manifests marked tissue segregation.

## Appendix Supplementary material

Table S1 Reported pathogenic variants in the *MT-TW* gene (n = 16). B = blood (leucocyte), Bu = buccal smear, CPEO = chronic progressive external ophthalmoplegia, F = fibroblast, H = hair follicle, HCM = hypertrophy cardiomyopathy, LA = lactic acidosis, M = skeletal muscle, MM = mitochondrial myopathy, OA = optic atrophy, n.d. = not done, RP = retinitis pigmentosa, U = urinary epithelial cell. Table S1
