# Supplementary material for: Clinical features of the pathogenic m.5540G>A mitochondrial transfer RNA tryptophan gene mutation
Source: Neuromuscul Disord. 2016 Oct;26(10):702–5. doi: 10.1016/j.nmd.2016.08.009 (PMC5066368; doi:10.1016/j.nmd.2016.08.009)
Supplement: Table S1 — Reported pathogenic variants in the MT-TW gene (n = 16). B = blood (leucocyte), Bu = buccal smear, CPEO = chronic progressive external ophthalmoplegia, F = fibroblast, H = hair follicle, HCM = hypertrophy cardiomyopathy, LA = lactic acidosis, M = skeletal muscle, MM = mitochondrial myopathy, OA = optic atrophy, n.d. = not done, RP = retinitis pigmentosa, U = urinary epithelial cell. [file mmc1.docx]

| **Pathogenic variant** | **Clinical phenotype** | **Heteroplasmy** | | | | **Inheritance** | **Reference** |
| --- | --- | --- | --- | --- | --- | --- | --- |
|  |  | **M** | **B** | **U** | **F** |  |  |
| m.5521G>A | Adult-onset MM | 98% | 0% | n.d. | n.d. | Possible maternal | [1] |
| m.5522G>A | MM | 76% | 5% | 11% | n.d. | Possible *de novo*, not detectable in maternal tissues (B, U) | [2] |
| m.5523T>G | Leigh syndrome | n.d. | 66% | n.d. | n.d. | Undetermined | [3] |
| m.5532G>A | Neurogastrointestinal syndrome, CPEO | 92% | 21% | n.d. | 37% | Maternal, detectable in unaffected maternal and brother’s blood (7% and 9% respectively) | [4] |
| m.5537insT | Leigh syndrome, OA, LA | >92% | >92% | n.d. | n.d. | Maternal, detectable in multiple members | [5] |
| m.5538G>A | Myoclonic epilepsy | 65% | 5% | 5% | n.d. | Maternal, mother with diabetes (M 20%) | [6] |
| m.5540G>A | Epilepsy, ataxia, RP, renal, stroke | 72% | 12% | 34% | n.d. | *De novo* | Present case, [7, 8] |
| m.5541C>T | MELAS | 84% | n.d. | 87% | n.d. | Maternal, unaffected mother’s urine (51%) | [9] |
| m.5543T>C | MM | 95% | 0% | n.d. | 0% | Possible *de novo*, not detectable in unaffected mother’s blood | [10] |
| m.5545C>T | HCM, LA, chorea, proximal weakness, seizure, ataxia | 25% | 13% | n.d. | 17% | *De novo*, not detectable in maternal tissues (B, U, H, Bu) | [11] |
| m.5549G>A | Dementia, chorea, deafness, ataxia | 86% | 40% | n.d. | n.d. | Undetermined | [12] |
| m.5556G>A | Encephalomyopathy, West syndrome | 93% | n.d. | n.d. | 92% | Possible *de novo*, not detectable in mother’s blood | [13] |
| m.5556G>C | Adult-onset encephalomyopathy | 70% | 0% | 0% | n.d. | *De novo*. Not detectable in elder brother’s tissues (M, B) | [14] |
| m.5559A>G | Leigh syndrome | n.d. | 43% | n.d. | n.d. | Maternal, mother’s blood (23%) | [3] |
| m.5567T>C | Adult onset MM | 50% | n.d. | 40% | 30% | Possible *de novo*, not detected in sister’s blood | [15] |

**Supplemental Table. Reported pathogenic variants in the *MT-TW* gene (n=16).** B**=** blood (leucocyte), Bu= buccal smear, CPEO= chronic progressive external ophthalmoplegia, F= fibroblast, H= hair follicle, HCM= hypertrophy cardiomyopathy, LA= lactic acidosis, M= skeletal muscle, MM= mitochondrial myopathy, OA= optic atrophy, n.d. = not done, RP= retinitis pigmentosa, U= urinary epithelial cell

**References**

[1] Silvestri G, Rana M, DiMuzio A, Uncini A, Tonali P, Servidei S. A late-onset mitochondrial myopathy is associated with a novel mitochondrial DNA (mtDNA) point mutation in the tRNA(Trp) gene. Neuromuscular disorders 1998;8:291-5.

[2] Baric I, Fumic K, Petkovic Ramadza D, et al. Mitochondrial myopathy associated with a novel 5522G>A mutation in the mitochondrial tRNA^Trp^ gene. Eur J Hum Genet 2013;21:871-5.

[3] Mkaouar-Rebai E, Chamkha I, Kammoun F, et al. Two new mutations in the MT-TW gene leading to the disruption of the secondary structure of the tRNA(Trp) in patients with Leigh syndrome. Mol Genet Metab 2009;97:179-84.

[4] Maniura-Weber K, Taylor RW, Johnson MA, et al. A novel point mutation in the mitochondrial tRNA^Trp^ gene produces a neurogastrointestinal syndrome. Eur J Hum Genet 2004;12:509-12.

[5] Santorelli FM, Tanji K, Sano M, et al. Maternally inherited encephalopathy associated with a single-base insertion in the mitochondrial tRNA^Trp^ gene. Ann Neurol 1997;42:256-60.

[6] Malfatti E, Cardaioli E, Battisti C, et al. A novel point mutation in the mitochondrial tRNA^Trp^ gene produces late-onset encephalomyopathy, plus additional features. J Neurol Sci 2010;297:105-8.

[7] Silvestri G, Mongini T, Odoardi F, et al. A new mtDNA mutation associated with a progressive encephalopathy and cytochrome c oxidase deficiency. Neurology 2000;54:1693-6.

[8] Granadillo JL, Moss T, Lewis RA, et al. Early Onset and Severe Clinical Course Associated with the m.5540G>A Mutation in. Mol Genet Metab Rep 2014;1:61-65.

[9] Blakely EL, Yarham JW, Alston CL, et al. Pathogenic mitochondrial tRNA point mutations: nine novel mutations affirm their importance as a cause of mitochondrial disease. Hum Mutat 2013;34:1260-8.

[10] Anitori R, Manning K, Quan F, et al. Contrasting phenotypes in three patients with novel mutations in mitochondrial tRNA genes. Molecular genetics and metabolism 2005;84:176-88.

[11] Sacconi S, Salviati L, Nishigaki Y, et al. A functionally dominant mitochondrial DNA mutation. Hum Mol Genet 2008;17:1814-20.

[12] Nelson I, Hanna MG, Alsanjari N, Scaravilli F, Morgan-Hughes JA, Harding AE. A new mitochondrial DNA mutation associated with progressive dementia and chorea: a clinical, pathological, and molecular genetic study. Ann Neurol 1995;37:400-3.

[13] Smits P, Mattijssen S, Morava E, et al. Functional consequences of mitochondrial tRNA^Trp^ and tRNA^Arg^ mutations causing combined OXPHOS defects. Eur J Hum Genet 2010;18:324-9.

[14] Sanaker PS, Nakkestad HL, Downham E, Bindoff LA. A novel mutation in the mitochondrial tRNA for tryptophan causing a late-onset mitochondrial encephalomyopathy. Acta Neurol Scand 2010;121:109-13.

[15] Valente L, Piga D, Lamantea E, et al. Identification of novel mutations in five patients with mitochondrial encephalomyopathy. Biochim Biophys Acta 2009;1787:491-501.
